# Trajectory of pain threshold and its association with acute pain after thoracic surgery: a prospective observational study

**DOI:** 10.1186/s13019-023-02424-w

**Published:** 2023-11-14

**Authors:** Fei Wang, Meng-Chan Ou, Yi-Hao Zhu, Tao Zhu, Xue-Chao Hao

**Affiliations:** 1 Department of Anesthesiology, Research Units of Perioperative Stress Assessment and Clinical Decision(2018RU012), West China Hospital of Sichuan University, Chinese Academy of Medical Sciences, Chengdu, 610041 China; 2 Translational Neuroscience Center, West China Hospital of Sichuan University, Chengdu, 610041 China; 3 Department of Anesthesiology, Sichuan Provincial People’s Hospital, School of Medicine, University of Electronic Science and Technology of China, Chengdu, China; 4 Department of Anesthesiology, Sichuan Clinical Research Center for Cancer, Sichuan Cancer Hospital & Institute, Sichuan Cancer Center, Affiliated Cancer Hospital of University of Electronic Science and Technology of China, Chengdu, China

**Keywords:** Analgesia, Pain threshold, Analgesia, patient-controlled, Pain, postoperative

## Abstract

**Background:**

Postoperative analgesic management is an ongoing challenge. The pain threshold (PT) is an objective index that reflects the body’s sensitivity to pain and can be used for quantitative pain assessment. We hypothesized that the PT is correlated with postoperative pain and can thus be used to guide postoperative pain management.

**Methods:**

This study involved 93 patients who underwent thoracoscopic surgery from December 2019 to February 2020. The PT was measured with transcutaneous electrical stimulation before surgery (T_0_) and at 1 h (T_1_), 6 h (T_6_), and 24 h (T_24_) after surgery. The visual analogue scale (VAS) score was used to evaluate the severity of postoperative pain at the same time. The PT variation (PTV) after surgery was calculated as the ratio of the postoperative PT to preoperative PT.

**Results:**

The postoperative PT was higher than the preoperative PT and showed a downward trend within 24 h after surgery; the PTV also showed a downward trend within 24 h after surgery. PT-T_1_ was negatively correlated with VAS-T_1_ at rest and during motion (rest: VAS-T_1_*r* = − 0.274, *P* = 0.008; motion: VAS-T_1_*r* = − 0.298, *P* = 0.004). PTV-T_1_ was negatively correlated with VAS-T_1_ during motion (*r* = − 0.213, *P* = 0.04). Lower VAS-T_1_ scores (< 4) at rest and during motion were associated with higher PT-T_1_ (rest: *t* = 2.452, *P* = 0.016; motion: *t* = 2.138, *P* = 0.035). The intraoperative sufentanil dose was associated with a postoperative increase in PTV-T_1_. Increased rescue analgesic administration was associated with PTV elevation. However, the incidence of dizziness in patients with moderate PTV-T_24_ was lower than that in patients with high or low PTV-T_24_ (*χ*^*2*^ = 8.297, *P* = 0.015).

**Conclusions:**

The postoperative PT was higher than the preoperative PT and showed a downward trend within 24 h after surgery; PTV also showed a downward trend within 24 h after surgery. The PT and PTV were negatively correlated with the pain intensity at rest and during motion and were associated with perioperative analgesic consumption and the incidence of adverse events.

## Background

Approximately 86% of patients experience pain after surgery, and 75% have moderate to extreme pain [1]. Poorly controlled acute postoperative pain delays the recovery time, prolongs the hospital stay, and increases medical expenses [2]; thus, it remains a major clinical challenge [3]. Controlling postoperative pain to achieve a balance between sufficient analgesia and few adverse reactions is the key to effective postoperative functional rehabilitation including deep breathing and effective coughing, especially in patients undergoing thoracic surgery [4].

Because of the dynamic effects of intraoperative and postoperative analgesics and the incision healing process, the profile of pain sensitivity and severity is time-dependent and individualized. A recent meta-analysis showed that acute intense postoperative pain after total hip arthroplasty resolved by 4 to 6 h after surgery in most patients [5]. Evaluation of analgesic effects and adjustment of pain management programs have been recommended [3]. Therefore, accurate methods to evaluate the postoperative pain profile are needed to establish a dynamic analgesia regimen. The pain threshold (PT) obtained by electrical stimulation is an objective index that reflects the body’s sensitivity to pain and can be used for quantitative pain assessment.

The visual analogue scale (VAS) and numeric rating scale (NRS) are mainly used to evaluate the severity of pain. These methods primarily rely on patients’ subjective feeling, which is determined by comparison with the hypothetical value of “maximum pain intensity” [6]. Patients’ sensitivity to pain is reportedly associated with the severity of postoperative pain and can be modulated by analgesics [7]. Pain sensitivity is effectively measured by detecting the PT induced by mechanical pressure or an electrical stimulus [8]. The advantage of an electrical stimulus is that it is convenient and repeatable [9]. The electric PT is the minimum current at which a patient feels pain and is measured mainly by stimulating the patient’s skin with an electrical stimulation device; the result is electronically recorded [10]. The results of previous studies have indicated that the electrical PT can reflect patients’ sensitivity to pain [10].

Previous studies have also shown that the preoperative PT is associated with postoperative pain in patients undergoing knee arthroplasty [11] and obstetrics and gynecologic surgery [12, 13]. According to the mode of stimulation, varying ability of preoperative quantitative sensory testing to predict postoperative pain has been reported [14]. Whether a lower PT before surgery contributes to greater postoperative pain remains controversial [7, 15]. Notably, the PT is regulated in a dual-directional manner during or after exposure to injury, opioids, or ketamine [8, 16]. The trajectory of the perioperative PT is unclear. Elucidating the trajectory of the perioperative PT may be helpful to understand the dynamic path of postoperative pain severity and the effects of postoperative analgesia.

Therefore, the present study was performed to explore [1] the trajectory of the PT and its association with postoperative pain and [2] the factors contributing to perioperative changes in the PT, the correlation between PT variation (PTV), and the incidence of postoperative adverse events in patients undergoing video-assisted thoracoscopic surgery.

## Methods

This prospective observational study was conducted at West China Hospital of Sichuan University. The protocol was approved by the ethics committee of Sichuan University (approval no. 2019836) and registered at www.chictr.org.cn (ChiCTR1900028218) on 15 December 2019. The participants were enrolled from 25 December 2019 to 20 February 2020, and all provided written informed consent.

### Inclusion and exclusion criteria

The inclusion criteria were an age of 18 to 75 years, American Society of Anesthesiologists physical status of I or II, and scheduled video-assisted thoracoscopic surgery under general anesthesia. The exclusion criteria were refusal to participate in the study; a history of chronic pain or use of analgesics for > 1 month; use of opioids or other analgesics within 24 h before surgery; a plan to perform delayed extubation after surgery; allergy to opioids or inability to operate a patient-controlled analgesia (PCA) device; a history of drug or alcohol abuse, psychiatric disorder, hearing or speech impairment, or neuromuscular diseases; body mass index of > 35 or < 18 kg/m^2^; and sensory abnormalities, dermatitis, or skin lesions at the test site.

### PT measurement

**Fig. 1:**
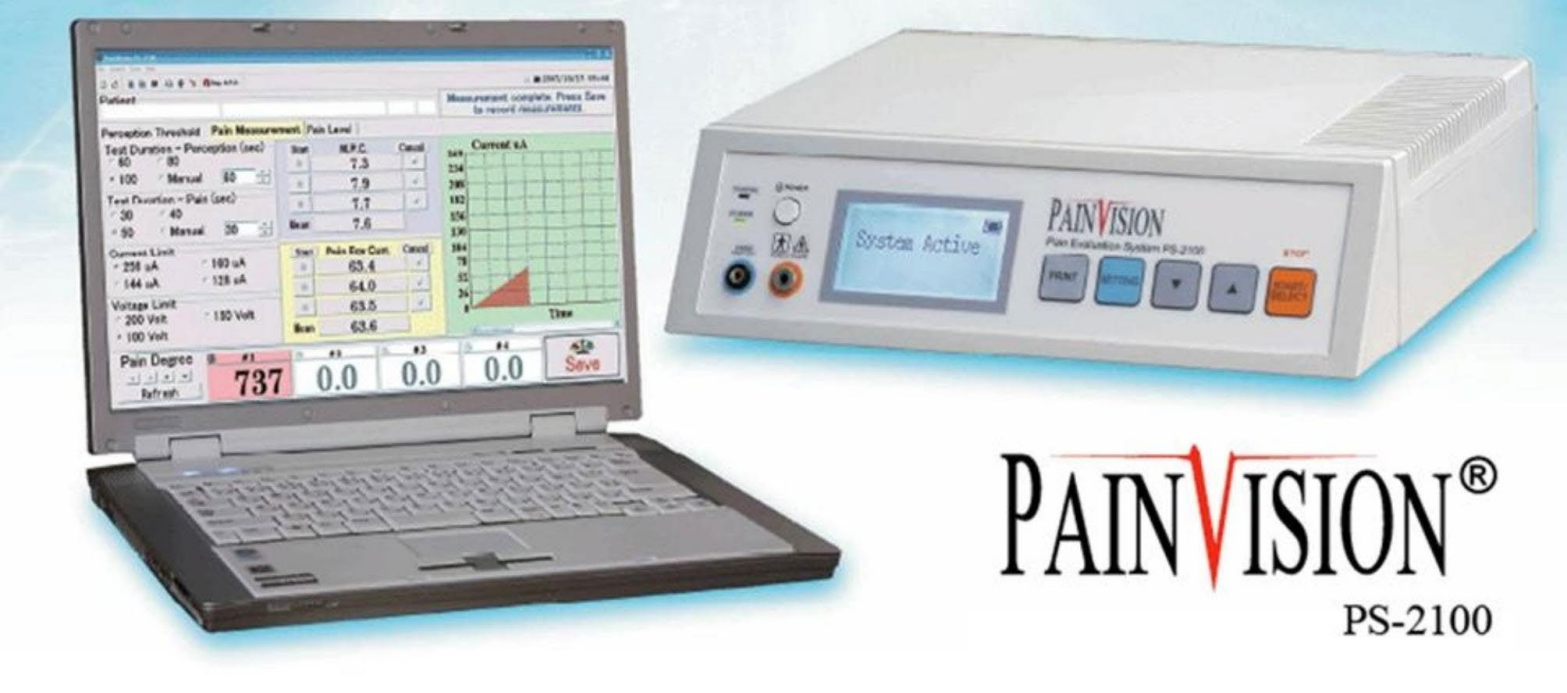
Pain Vision™ PS-2100 (Nipro Corporation, Osaka, Japan)

The day before surgery, the preoperative PT was measured and the patients were instructed on use of the 10-cm VAS for pain assessment (0 = no pain to 10 = worst pain), the necessity and main points of effective coughing, and the procedure of PT testing. The PT was measured using a PainVision PS-2100 (Nipro Corporation, Osaka, Japan) (Fig. 1), which had been used for evaluation of pain in a previous study [17]. After an electrode was attached to the ulnar side of the non-dominant forearm, the patient was instructed to close their eyes during the procedure and press a button as soon as pain was perceived. An electrical stimulus with continuously increasing intensity was delivered, and the stimulus intensity at which the patient perceived pain was recorded. The PT was defined as the smallest current value (µA) that was perceived as painful. Three detection tests at intervals of 1 min were administered, and the mean PT was calculated.

The PT was measured repeatedly in the same way at 1, 6, and 24 h after surgery, and the VAS scores at rest and during coughing were also assessed at these time points. The PT was measured before the VAS scores. The PT and VAS were measured by the same experienced investigator. With respect to rescue analgesia, patients with a VAS score of > 4 at rest or cough-restricted pain were given intravenous or intramuscular dezocine (5 mg) or pressed the PCA button by themselves, and the number of rescue analgesic events was recorded.

PTV was calculated in the present study because it adjusted the individual variation of the baseline PT and provided a dimensionless variable that could be obtained by multiple approaches, regardless of the type of stimulation (cold, pressure, or electrical current). PTV after surgery was calculated as the ratio of the postoperative PT to preoperative PT as follows:

PTV = postoperative PT / baseline PT × 100%.

### Anesthesia protocol

The patients did not receive premedication before anesthesia. They were routinely monitored using electrocardiography, pulse oximetry, noninvasive arterial blood pressure, and a bispectral index monitor (Covidien/Medtronic, Minneapolis, MN, USA) in the operating room. General anesthesia was induced by intravenous propofol 1.5 to 2.5 mg/kg, sufentanil 3 µg/kg, and cisatracurium 0.2 mg/kg. Anesthesia was maintained with intravenous remifentanil infusion and desflurane inhalation. A target bispectral index of 40 to 60 was maintained by adjusting the concentration of desflurane. The initial dosage of remifentanil was 0.1 µg·kg^− 1^·min^− 1^ and was regulated in a stepwise manner by increments of 0.05 µg·kg^− 1^·min^− 1^ to achieve a heart rate of 60 to 80 beats/min and a blood pressure that was ± 20% of the pre-induction value. Sufentanil was administered at a dose of 0.1 µg/kg prior to commencement of the skin incision and 15 min prior to the expected conclusion of surgery. At skin closure after surgery, the desflurane and remifentanil were terminated, and a PCA device was applied to the patients for postoperative analgesia with sufentanil (0.03 µg·kg^− 1^·h^− 1^) and dexmedetomidine (2 µg/h). The patients were discharged from the post-anesthesia care unit after a modified Aldrete score of ≥ 9 was obtained.

### Data collection

The patients’ baseline data were recorded, including their demographic information, perioperative PT, American Society of Anesthesiologists physical status, and type of surgery. Intraoperative data included the duration of anesthesia and surgery and the doses of sufentanil and remifentanil. The postoperative data included the postoperative PT, VAS score, sufentanil consumption by PCA, number of PCA presses, number of postoperative rescue analgesics, Ramsay score (1 = anxious, agitated, or restless; 2 = cooperative, orientated, or tranquil; 3 = responds to commands only; 4 = brisk response; 5 = sluggish response; and 6 = no response), and occurrence of adverse events (e.g., nausea and vomiting, dizziness, urinary retention, respiratory depression, or skin itching). Respiratory depression was defined as a respiratory rate of < 10 breaths/min or oxygen saturation of < 92% for > 5 min.

The primary outcome was the correlation between the PTV and VAS score. The secondary outcomes were the pain threshold and the postoperative pain VAS score at 1 h, 6 h, 24 h after surgery, factors contributing to the perioperative change of pain threshold, and the correlation between PTV and the incidence of postoperative adverse events.

### Statistical analysis

At least 92 patients were required to obtain a correlation coefficient of 0.33 between PTV and the VAS scores. This calculation was based on a type-I error of 0.05 and power of 0.9 for two-tailed analysis. To account for patients who may be excluded because of alterations in surgical procedures or analgesic regimens, we aimed to recruit a total of 100 patients.

A biomedical statistician performed a statistical review of the study before submission for peer review. All statistical analyses were carried out using SPSS software ver. 23.0 (IBM Corp., Armonk, NY, USA), GraphPad Prism ver. 8.0 (GraphPad Software, San Diego, CA, USA), or Origin 2019 (OriginLab, Northampton, MA, USA). Data normality was checked with the Kolmogorov–Smirnov test, and continuous variables are presented as mean ± standard deviation or median (interquartile range). We modeled PTV using a logarithmic transformation to reduce the potential effect of extreme values within its highly skewed distribution. The postoperative PT and PTV at each time point were analyzed with repeated-measures analysis of variance (ANOVA). Postoperative VAS-T_1_, VAS-T_6_, and VAS-T_24_ were analyzed by the Friedman test. Spearman correlation analysis was used to estimate the relationship of the PT and PTV with the VAS score at each time point after surgery. The PT and PTV were compared between the mild pain group (VAS score of < 4, M group) and the moderate to severe pain group (VAS score of ≥ 4, MS group) at each time point after surgery using an independent-samples t test. Perioperative PTV was divided into low, moderate, and high groups according to the levels of PTV at each time point after surgery. The incidence of adverse effects and the proportion of patients requiring rescue analgesia was compared between the low, moderate, and high PTV groups at each time point by Pearson’s chi-square test or Fisher’s exact test. Multiple linear regression analysis was used to analyze the influencing factors of PTV. Collinearity statistics were used to estimate the risk of collinearity in the multivariate analysis. *P*-values were two-tailed, and *P*-values of < 0.05 were considered statistically significant. Effect sizes with a 95% confidence interval were calculated if necessary.

## Results

### Patient characteristics and postoperative analgesia

**Fig. 2:**
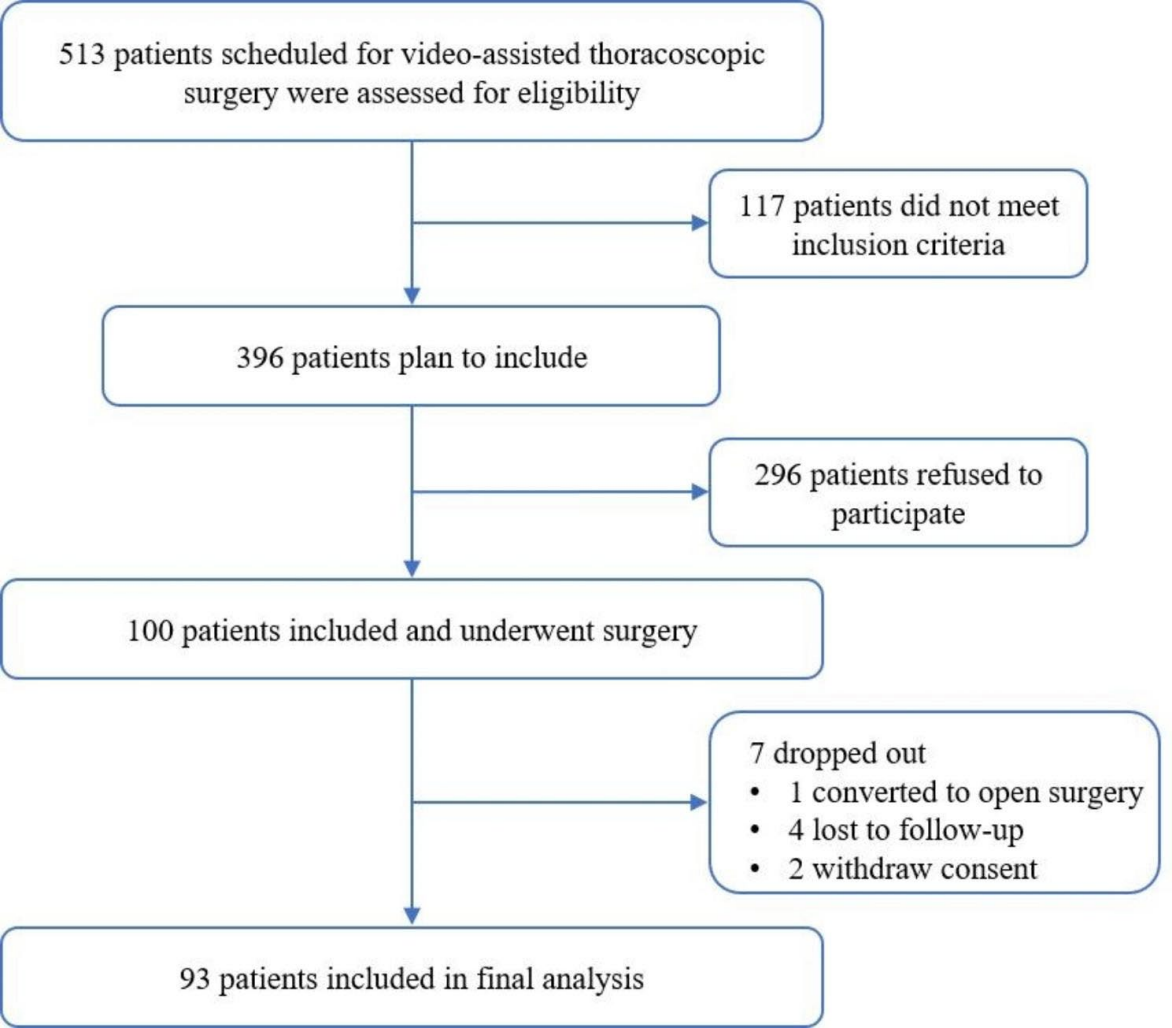
Schematic illustration of the present study

After screening, 100 eligible patients were recruited, and a total of 93 patients were included in the final analysis after excluding 7 patients who dropped out (1 patient was converted to open surgery, 4 patients were lost to follow-up, and 2 patients stopped using PCA) (Fig. 2). The patients’ clinical characteristics and information regarding anesthesia, surgery, and postoperative analgesia are shown in Tables 1 and 2. The patients comprised 38 (40.9%) men and 55 (59.1%) women with a mean age of 55.03 ± 8.76 years. The incidence of moderate to severe pain (VAS score of ≥ 4) at rest and during motion were 38.7% and 69.9% at 1 h, 22.6% and 69.9% at 6 h, and 22.6% and 63.4% at 24 h, respectively.

**Table 1:** Characteristic variables of the patients, anesthesia and surgery. Data are presented as the mean ± SD, median (interquartile ranges) or n (%)

Variables	
Ages (yr)	55.03 ± 8.76
Sex (male/female)	38/55
Weight (kg)	59.12 ± 10.57
Height (m)	1.60 ± 0.078
BMI (kg/m²)	22.85 ± 2.82
ASA physical status (I/II)	7/86
Medical conditions	
Hypertension, n (%)	8(8.6%)
Diabetes mellitus, n (%)	2(2.1%)
Preoperative PT (µA)	40.9(30.8–59.2)
Type of surgery	
Pulmonary lobectomy	70(75.3%)
Pulmonary segmentectomy	19(20.4%)
Mediastinal tumor resection	3(3.2%)
Plate removal for funnel chest repair surgery	1(1.1%)
Duration of anaesthesia (min)	146.12 ± 39.46
Duration of surgery (min)	93.73 ± 34.14
Intra-operative sufentanil consumption (ug)	32.18 ± 5.56
Intra-operative remifentanil consumption (ug·kg^− 1^·min^− 1^)	0.10 ± 0.01

**Table 2:** Description of PT, PTV, VAS and Ramsay score. Data are presented as the mean ± SD or median (interquartile ranges)

Variables	T_0_	T_1_	T_6_	T_24_	*P* value
PT (µA)	40.9 (30.8–56.3)	86.1(68.5-106.6)*	65(51.5–82.7)*#	59.4(40.3–79.1)*#	< 0.001
PTV	-	2.02(1.54–2.75)	1.52(1.26–2.05)#	1.26(0.97–1.90)#	< 0.001
VAS at rest	-	3.0(0.5-5.0)	2.0(1.0–3.0)#	2.0(0.0–3.0)#	< 0.01
VAS at motion	-	5.0(3.0–6.0)	4.0(3.0–6.0)	4.0(3.0–6.0)	ns
The Ramsay score	-	2.64 ± 0.72	2.06 ± 0.29	2.00 ± 0	ns
*indicates *P* < 0.05, as compared with T_0_; #indicates *P* < 0.05, as compared with T_1_.					

### Perioperative trajectory of PT and PTV

**Fig. 3:**
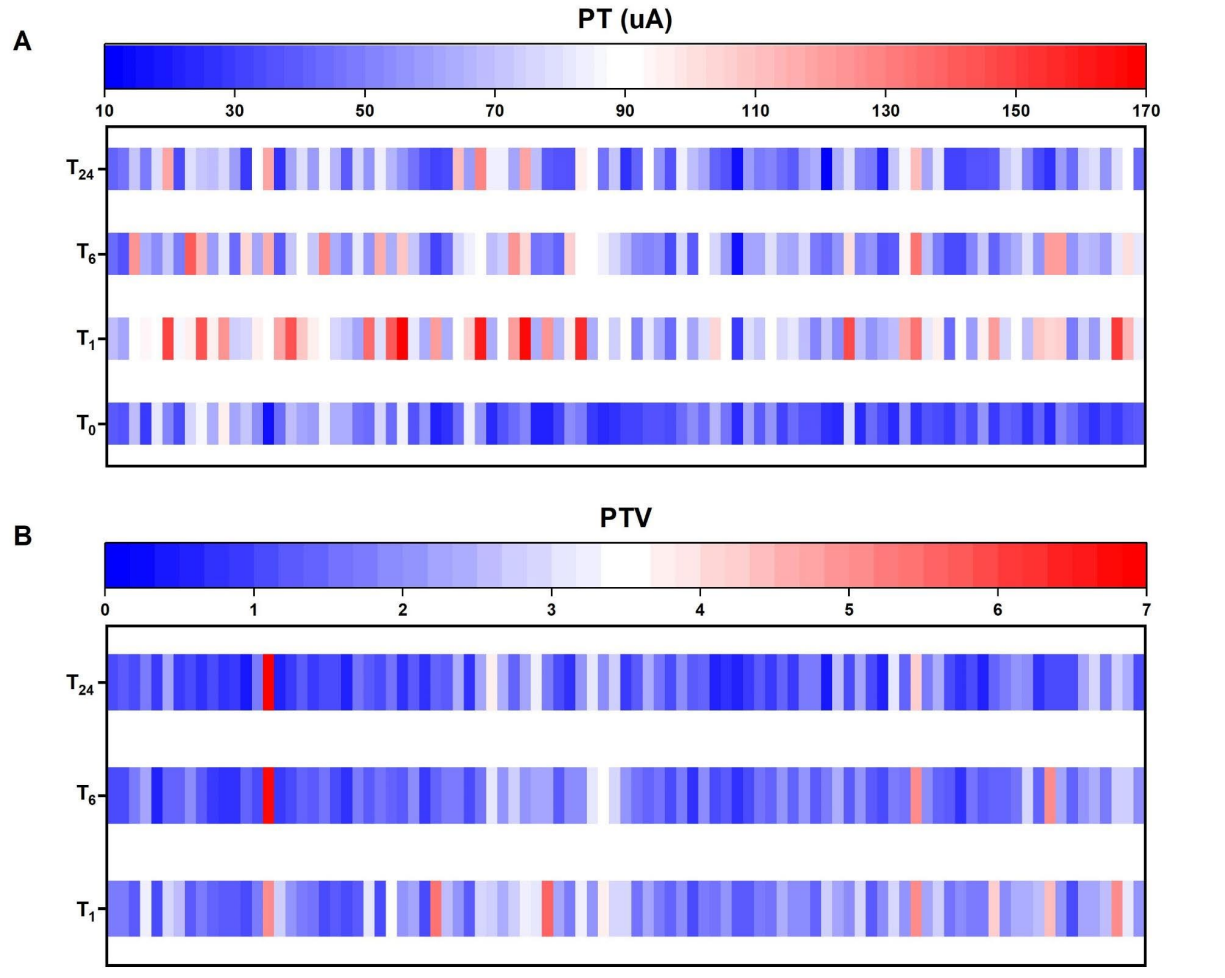
The heatmap analysis for tendency of perioperative PT, PTV. **A**, heatmap analysis of PT at each time point; **B**, heatmap analysis of PTV at each time point. Each bar indicates a patient, and the PT and PTV after surgery showed a downward trend in most patients

The postoperative PT was higher than the preoperative PT and showed a downward trend within 24 h after surgery, with statistical significance (*F* = 93.69, *P* < 0.001 by one-way repeated-measures ANOVA) (Fig. 3A; Table 2). The PTV and VAS score at rest also exhibited a downward trend within 24 h after surgery (Fig. 3A; Table 2). A statistically significant difference was found in the PTV and VAS scores at rest among T_1_, T_6_, and T_24_ (PTV, *P* < 0.001 by one-way repeated-measures ANOVA; VAS score, *P* = 0.003 by the Friedman test) (Table 2). The PT, PTV, and VAS score at rest at T_1_ were higher than those at T_6_ and T_24_ (Table 2). No significant difference was found in the Ramsay score or VAS score during motion at T_1_, T_6_, and T_24_ after surgery (Table 2).

### Relationship between perioperative PT, PTV, and VAS score

**Fig. 4:**
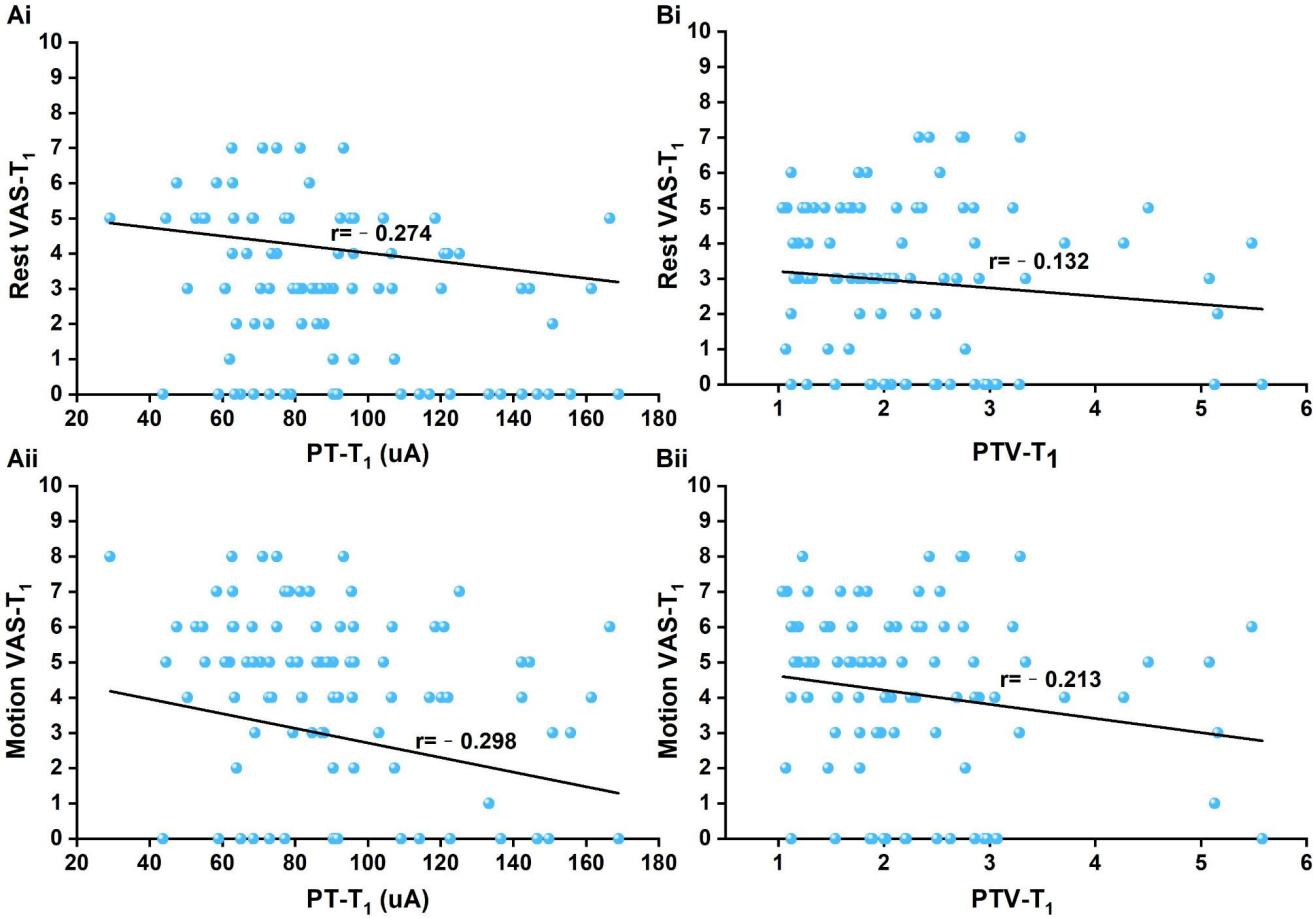
Scatter plot of postoperative PT-T_1_ and VAS rest and during motion

PT-T_1_ was negatively correlated with VAS-T_1_ at rest and during motion (rest: VAS-T_1_*r* = − 0.274, *P* = 0.008; motion: VAS-T_1_*r* = − 0.298, *P* = 0.004) (Fig. 4A). PTV-T_1_ was not correlated with VAS-T_1_ at rest (*P* = 0.131) but was negatively correlated with VAS-T_1_ during motion (*r* = − 0.213, *P* = 0.04) (Fig. 4B). Neither PT-T_6_ nor PTV-T_6_ was correlated with VAS-T_6_ at rest or during motion (PT: rest *P* = 0.203, motion *P* = 0.215; PTV: rest *P* = 0.917; motion *P* = 0.835). Neither PT-T_24_ nor PTV-T_24_ was correlated with VAS-T_24_ at rest or during motion (PT: rest *P* = 0.962, motion *P* = 0.258; PTV: rest *P* = 0.289, motion *P* = 0.309).

**Fig. 5:**
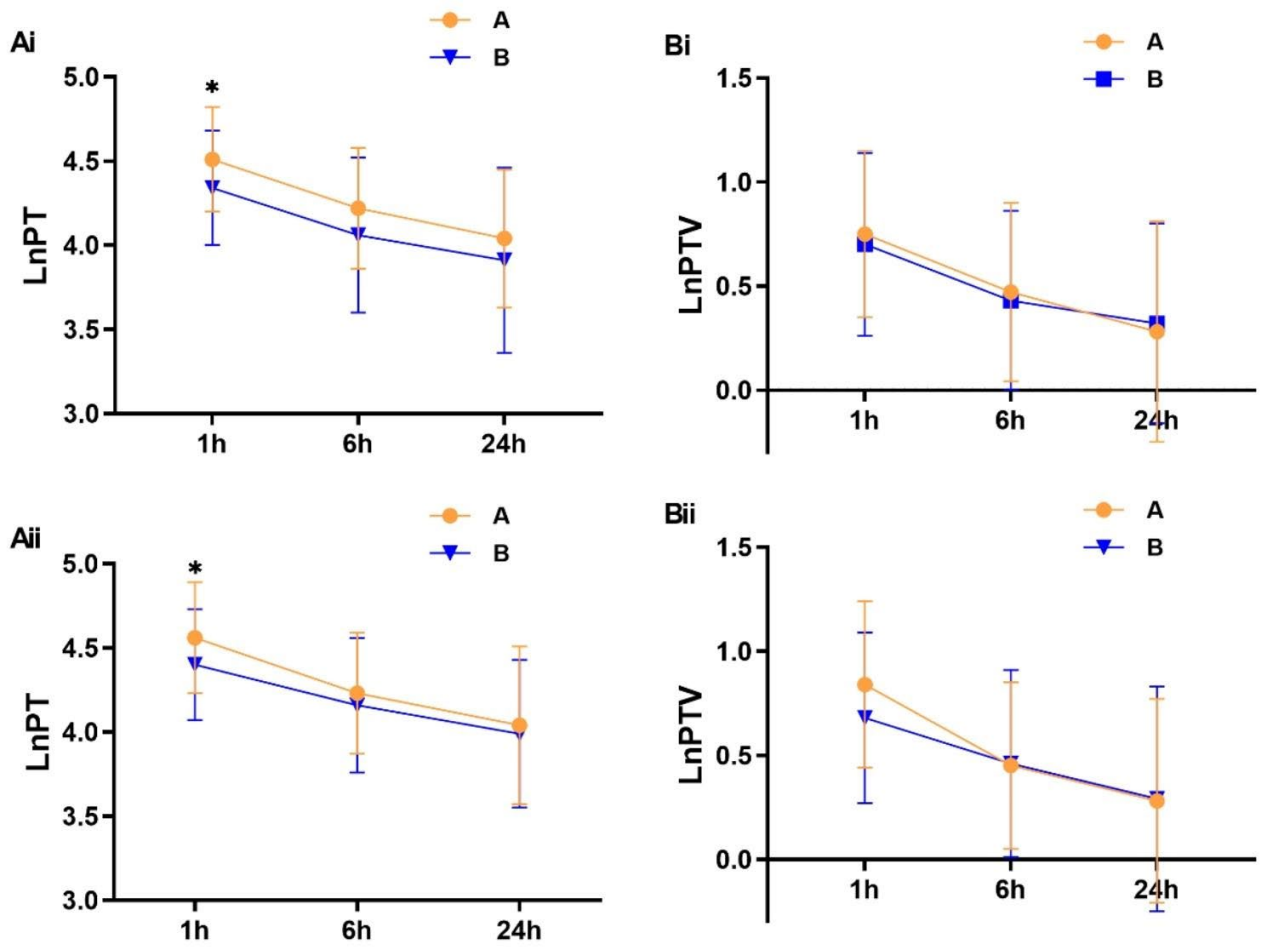
The PT and PTV of the patients between mild pain group (VAS < 4, Orange line **A**) and moderate to severe pain group (VAS ≥ 4, Bule line **B**) were compared at each time point after surgery. * indicated P < 0.05 by independent samples *t* test. LnPT: Logarithmic Pain Threshold, LnPTV: Logarithmic Pain Threshold Variation

Patients with mild pain (VAS score of < 4, M group) at rest and during motion showed a higher PT than those with moderate to severe pain (VAS score of ≥ 4, MS group) at T_1_ after surgery (rest: *t* = 2.452, *P* = 0.016; motion: *t* = 2.138, *P* = 0.035). There was no significant difference in PT-T_6_, PT-T_24_, PTV-T_1_, PTV-T_6_, or PTV-T_24_ (*P* > 0.05) between the M group and MS group at each time point after surgery (PT is shown in Fig. 5A and PTV is shown in Fig. 5B).

### Factors contributing to perioperative PTV

The multivariate analysis results showed that intraoperative sufentanil was an influencing factor of postoperative PTV-T_1_ (R^2^ = 0.103) (Table 3). The dosage of sufentanil in the PCA pump and dezocine within 24 h did not affect PTV-T_6_ or PTV-T_24_.

**Table 3:** Multivariate linear regression analysis to evaluate factors that influenced PTV-T_1_, PTV-T_6_, PTV-T_24_ postoperatively

Variables	PTV-T_1_			PTV-T_6_			PTV-T_24_			
	*Beta*	*t*	*P*	*Beta*	*t*	*P*	*Beta*	*t*	*P*	
Age	0.150	1.445	0.152	-0.024	-0.217	0.828	-0.089	-0.816	0.417	
Sex(man/woman)	-0.011	-0.105	0.917	0.041	0.352	0.726	-0.081	-0.654	0.515	
Intra-operative sufentanil dose (ug)	0.270	2.636	**0.010**	0.140	1.320	0.191	0.147	1.359	0.178	
Intra-operative remifentanil dose (ug)	0.066	0.613	0.542	0.150	1.303	0.196	0.058	0.487	0.627	
Sufentanil of PCA at 1 h after surgery (ug)	0.055	0.518	0.605	-	-	-	-	-	-	
Sufentanil of PCA at 6 h after surgery (ug)	-	-	-	-0.110	-0.922	0.359	-	-	-	
Sufentanil of PCA at 24 h after surgery (ug)	-	-	-	-	-	-	-0.149	-1.134	0.260	
Dezocine dose of 6 h after surgery (mg)	-	-	-	-0.020	-0.189	0.850	-	-	-	
Dezocine dose of 6-24 h after surgery (mg)	-	-	-	-	-	-	0.037	0.345	0.731	

### Relationship between PTV, rescue analgesia, and adverse events

**Fig. 6:**
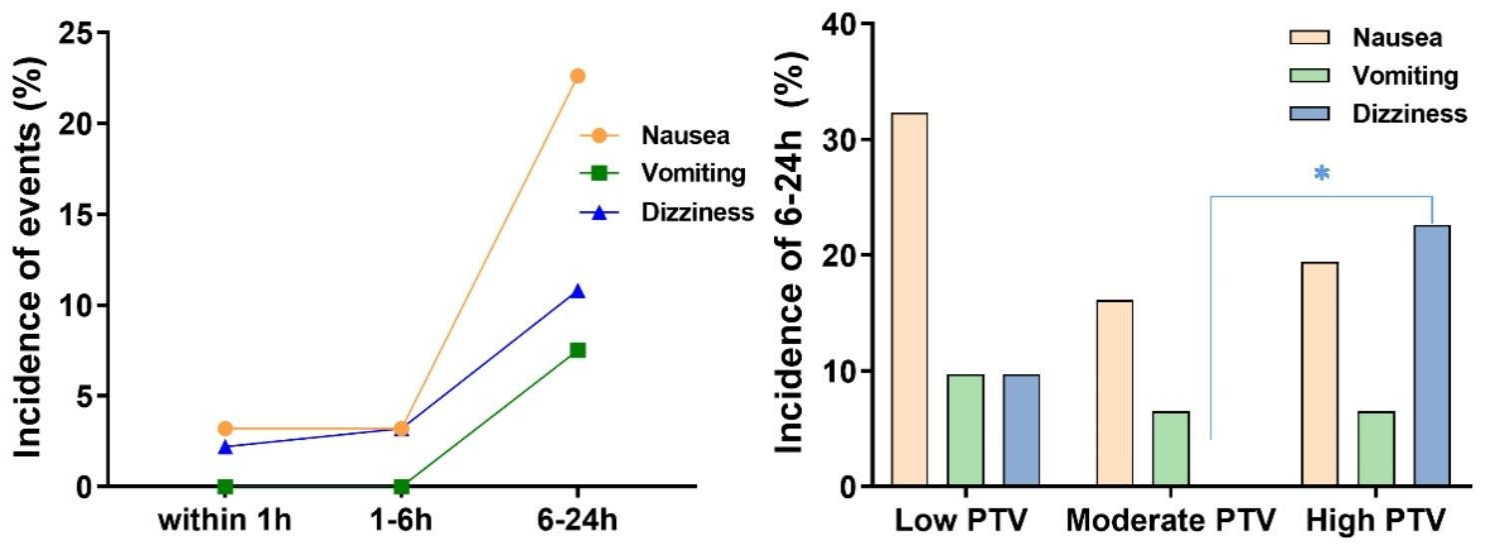
The incidence of nausea, vomiting, dizziness after surgery. **A**, Incidence of nausea, vomiting, and dizziness within 24 h after surgery; **B**, incidence of nausea, vomiting, dizziness in patients with low, moderate, high PTV-T_24_ group within 6-24 h after surgery. Data were expressed by ration (%). * indicated *P* = 0.015, by Fisher’s exact test

Within 24 h after surgery, the rate of rescue analgesia was 44.09%. The incidence of nausea, vomiting, and dizziness after surgery is shown in Fig. 6A. Perioperative PTV was divided into a low third, moderate third, and high third according to the levels of PTV at each time point after surgery. In total, 58.1% (18/31) of patients with high PTV-_24_, 41.9% (14/31) with moderate PTV-_24_, and 32.3% (10/31) with low PTV-_24_ received rescue analgesia within 6 to 24 h after surgery, but no significant difference was found (*χ*^*2*^ = 4.275, *P* = 0.118 by Pearson chi-square test). The incidence of dizziness in the moderate PTV-T_24_ group was lower than that in the high or low PTV-T_24_ group (*P* = 0.015 by Fisher’s exact test). No significant difference was found in the incidence of nausea and vomiting among the three groups (nausea: *χ*^*2*^ = 2.583, *P* = 0.379 by Pearson chi-square test; vomiting: *P* = 1.00 by Fisher’s exact test) (Fig. 6B).

## Discussion

In the present study, we explored the trajectory of the perioperative PT and PTV and the correlation between the PT or PTV with postoperative pain and adverse events. The results demonstrated that the postoperative PT was higher than the preoperative PT and showed a downward trend within 24 h after surgery. The extent to which the PT at 1 h after surgery increased was associated with intraoperative sufentanil consumption. The PT and PTV were negatively correlated with the postoperative pain VAS score, incidence of rescue analgesia, and adverse effects. Increased administration of analgesics achieved a higher PT or PTV but also more adverse events, suggesting an optimal PT or PTV for guiding postoperative pain management, especially for pain during motion. Pain threshold can be monitored and regulated by adjusting the analgesic dose to achieve adequate analgesia with few adverse effects.

### Measurement of PT

The PT was measured with transcutaneous electrical stimulation in the present study [18] because previous studies have shown that the electrical pain threshold is potentially valuable for reflecting pain sensitivity [14], convenient, and easy to use. Myelinated Aδ-fibers carry the signal more rapidly (5–30 m/s) than unmyelinated C-fibers (0.5–2.0 m/s), and Aδ-fibers primarily conduct the pain signal [19]. Thus, the PainVision device delivered an electrical sine wave stimulus (wave width of 0.3 ms) at a frequency of 50 Hz with a controllable linear increase in the stimulus intensity (0–200 µA). On the one hand, asepsis and convenience for repeat measurements are important. On the other hand, thin skin, a flat surface, a low distribution of sweat glands, and hair follicles lead to high sensitivity of electrical stimulation and good electrical conductance [19].

### Trajectory of PT and factors contributing to PTV

The present study demonstrated that the postoperative PT was higher than the preoperative PT and showed a downward trend within 24 h after surgery. The PTV also showed a downward trend within 24 h after surgery. The increase in the PT at 1 h after surgery was associated with greater intraoperative sufentanil consumption, and the downward trend in the PT and PTV might have been associated with the elimination of sufentanil and the incision healing process. However, a previous study demonstrated that the postoperative PT was significantly lower than that at baseline and returned to baseline after a long period [8]. This discrepancy was speculated to be associated with three main factors. First, different methods are used for PT measurement. The PT was measured by electrical stimulation in the present study, whereas hot and cold stimulation was used to measure the PT in several previous studies. Second, different intraoperative and postoperative analgesia regimens are used. Sufentanil and remifentanil were used for perioperative analgesia in the present study. Dexmedetomidine is among the pharmacological agents utilized for postoperative analgesic management, and the average dose of remifentanil was only 0.1 µg/kg/min in the present study. Previous studies have shown that use of high-dose remifentanil and fentanyl can result in hyperalgesia [16, 20]. However, recent studies have demonstrated that dexmedetomidine infusion can reduce opioid-induced hyperalgesia [21, 22]. Third, the timing of PT measurement differs among studies.

Considering that regional analgesic techniques may not be appropriate for all patients because of their coagulation function, PCA is a widely utilized, uncomplicated, and convenient method that empowers patients to manage their own pain [23]. A previous study [24] indicated that PCA may be an effective alternative to regional analgesia, and it has been recommended as a primary approach for pain management [25]. Hence, we explored the trajectory of pain changes under opioid-based PCA. However, considering the consensus between good clinical practice and scientific evidence, it is widely acknowledged that the implementation of preventive locoregional analgesic techniques can effectively enhance postoperative analgesia, minimize opioid consumption, and prevent the development of chronic pain [26]. We will sustain our efforts to improve the rate of multimodal analgesia in the perioperative period in accordance with the latest clinical guidelines in our practice. The trajectory of pain should be explored using different analgesic regimens.

The results of this study indicate that the PT or PTV can be controlled by adjusting the dosage of analgesics, helping to guide postoperative pain management. Higher intraoperative sufentanil consumption was positively associated with the PT at 1 h after surgery, and the patients who received more rescue analgesia within 6 to 24 h after surgery achieved higher PTV-T_24_. The results of the studies by Mauermann et al. [16, 27] and Wilder-Smith et al. [16, 27] were consistent with the results of this study, indicating that opioid use will increase the PT. A recent animal study also showed that dezocine, morphine, and nalbuphine all increased the electrical PT and thermal PT of rats [28]. The dosage of sufentanil in the PCA and dezocine within 24 h did not affect PTV-T_6_ or PTV-T_24_; the dezocine blood concentration peaked 10 to 90 min after administration, and the sufentanil speed was 0.03 µg·kg^− 1^·h^− 1^ in the PCA. At the time of PT measurement in the present study, the peak dezocine blood concentration might have already been reached and the speed of sufentanil administration in the PCA may have been too small to influence the PT.

We evaluated the relationship between the PT and analgesic administration, but the temporal pre- and post-relationship is unclear. It was difficult to evaluate whether administering analgesics increased the PT or whether patients with a high PT required rescue analgesics. PT was not measured in patients before and after rescue analgesia to clarify the relationship and trends. Patients who need rescue analgesics may have opioid-induced hyperalgesia. Other opiates can also lead to opioid-induced hyperalgesia. Some medications, such as dexmedetomidine, can reduce opioid-induced hyperalgesia [21]. The chosen analgesic protocol may impact the trajectory of pain. We examined the trajectory of pain by utilizing PCA as the primary analgesia method while taking into account confounding factors and surgeon cooperation factors. Further research is recommended to investigate the potential alterations in the pain trajectory under multimodal analgesia. Additionally, the causal relationship between analgesics and the PT should be further explored through randomized controlled trials.

### Correlation between PT, PTV, VAS score, and adverse effects

The preoperative PT can reportedly predict postoperative pain in patients undergoing cesarean section and total knee replacement [10, 29]. However, little is known about the relationship between the PT and pain severity at a given time point after surgery. The present study showed that PT-T_1_ was negatively correlated with VAS-T_1_ at rest and during motion and that PTV-T_1_ was negatively correlated with VAS-T_1_ during motion. However, the correlation between PT-T_1_ or PTV-T_1_ and VAS-T_1_ was not strong. Notably, previous studies of groin hernia repair in men and percutaneous nephrolithotomy showed no significant predictive role for the PT [30, 31].

The present study showed no significant correlation between postoperative PT-T_6_, PT-T_24_, PTV-T_6_, PTV-T_24_, and the VAS score at corresponding time points. This might be explained by the non-linear distribution of variables and the different analgesics used after surgery [32]. The use of more rescue analgesics within 6 to 24 h after surgery is associated with higher PTV-T_24_, suggesting that PTV is potentially related to postoperative pain.

Although the PT and PTV are negatively correlated with the VAS score, aiming for infinite increases in the PT and PTV is not appropriate when designing postoperative analgesia regimens. In the present study, the incidence of dizziness within 6 to 24 h was significantly lower in the moderate PTV-T_24_ group than in the low and high PTV-T_24_ groups. Additionally, the incidence of nausea within 6 to 24 h was lower in the moderate PTV-T_24_ group than in the low and high PTV-T_24_ groups. These findings indicate that moderate PTV may be a potential objective target range for guiding postoperative pain management, especially for the guidance and management of pain during motion, which needs to be further explored.

### Significance of PTV

Two main factors were considered in our calculation of PTV in the present study. First, previous studies have proven that the baseline PT before surgery is affected by numerous perioperative factors, such age, sex, and affective disorders [33, 34]. Second, PTV may eliminate the effect of confounding factors among individuals and provide a dimensionless variable that can be obtained by multiple approaches regardless of the type of stimulation (cold, pressure, or electrical current). An appropriate range of PTV may be an optimal target for postoperative analgesia based on the integration of our analysis results regarding pain intensity and adverse events from three time points after surgery.

This study had several limitations. First, the postoperative analgesic regimen was based on analgesics with systemic effects. The findings of our study are not applicable to patients who are administered local anesthetics. Second, although the sample size was estimated before the study commenced, a low level of correlation was obtained because of the non-linear or non-normal distribution of variables. Third, the postoperative PT was measured at 1, 6, and 24 h after surgery with the aim to avoid measurement in evening. More intensive and extended measurement times may provide more information regarding PT trajectory dynamics. Fourthly, the repeated analysis involves the problem of multiplicity of P-values. The results need to be interpreted with caution and confirmed by further studies.

## Conclusions

The PT increased after surgery and showed a downward trend within 24 h after surgery. The PT and PTV were negatively correlated with the pain intensity and were associated with perioperative analgesic consumption and the incidence of adverse events. The results of the present study provide additional features of the postoperative pain profile and potential instruction for choosing an optimal analgesic regimen for different phases after surgery.

## Data Availability

No additional data are available.

## Abbreviations

PT: Pain Threshold
PTV: Pain Threshold Variation
VAS: Visual Analogue Scale
NRS: Numeric Rating Scale
PCA: Patient-Controlled Analgesia
